# Behçet’s Disease with Intestinal Involvement can be Distinguished from Inflammatory Bowel Diseases by Measurement of Common Femoral Vein Wall Thickness

**DOI:** 10.31138/mjr.120824.dfh

**Published:** 2025-05-28

**Authors:** Gizem Sevik, Rabia Ergelen, Ilkay Ergenc, Efe Soydemir, Fatma Temiz, Ozlen Atug, Haner Direskeneli, Fatma Alibaz-Oner

**Affiliations:** 1 Division of Rheumatology, Department of Internal Medicine, School of Medicine, Marmara University, Istanbul, Turkey;; 2 Department of Radiology, School of Medicine, Marmara University, Istanbul, Turkey;; 3 Division of Gastroenterology, Department of Internal Medicine, Marmara University, School of Medicine, Istanbul, Turkey; 4 Department of Internal Medicine, School of Medicine, Marmara University, Istanbul, Turkey

**Keywords:** Behçet’s disease, Crohn’s disease, ulcerative colitis, inflammatory bowel disease, common femoral vein wall thickness

## Abstract

**Objective/Aim:**

Differentiating the gastrointestinal (GI) involvement of Behçet’s disease (BD) and inflammatory bowel diseases (IBD) can be a diagnostic challenge. We previously reported that the wall thickness of the common femoral vein (CFV) is higher in BD patients compared to Crohn’s disease (CD) with a limited number of IBD patients with only CD. This study aimed to evaluate the CFV thickness measurement in BD patients and in a larger group of IBD patients including both ulcerative colitis (UC) and CD.

**Methods:**

The study included patients with BD (n=117), IBD (n=87, [53 CD, 34 UC]), and healthy gender-matched controls (HC) (n=85). CFV wall thicknesses were measured with Doppler ultrasonography.

**Results:**

Among BD patients, 70 (59.8%) had major organ (48[41.0%] vascular, 21[30.0%] ocular, 11[15.7%] gastrointestinal, and 8[11.4%] neurological) involvement. The mean right CFV thickness was 0.75±0.21 mm, 0.32±0.08 mm, and 0.28±0.13 mm for BD, IBD, and HC, respectively (p<0.001). BD patients had significantly higher CFV wall thicknesses compared to IBD patients and HC (adj. p<0.001 for both), and CFV thicknesses in IBD were comparable to HC (adj.p>0.05). Among BD patients, CFV thicknesses did not differ in patients with and without GI involvement. CFV thicknesses were also similar in patients with CD and UC.

**Conclusion:**

CFV thickness was significantly higher in BD compared to CD and UC patients. These results suggest that the CFV wall thickness measurement may be used in daily practice to differentiate GIBD from IBD.

## INTRODUCTION

Behçet’s disease (BD) is an inflammatory disease characterised by oral aphthous ulcers, skin and ocular lesions, and involvement of the vascular, gastrointestinal (GI), or neurological systems.^1^ The GI involvement of BD (GIBD) may present as diarrhoea, abdominal pain, bleeding, fistula, or perforation, and these can also be the initial signs of inflammatory bowel disease (IBD).^2–4^ BD-like extraintestinal manifestations, including oral ulcers, erythema nodosum, arthritis, or uveitis are also seen in 25–40% of IBD patients.^5^

Ischemic changes related to vasculitis cause higher rates of major GI bleeding and GI perforation in patients with GIBD than IBD.^6^ Although GI fistula may be seen in GIBD, fistulising perianal disease is strongly associated with Crohn’s disease (CD).^7,8^ However, histopathologic or macroscopic inflammatory changes in GIBD are similar to CD. As a result, especially in patients with nonspecific GI symptoms, differentiating GIBD from IBD can be a diagnostic challenge in clinical practice.

Our group previously reported that increased wall thickness of the common femoral vein (CFV) measured by Doppler ultrasonography (US) is a distinctive feature of BD, with nearly 80% sensitivity and specificity.^9^ Further studies from Turkey also showed similar results to our study.^10–13^ Furthermore, we assessed the utility of the measurement of CFV wall thickness in differentiating BD and CD and found significantly higher CFV thickness in BD compared to CD patients, however, ulcerative colitis (UC) patients were not evaluated.^14^ In this study, we aimed to evaluate the CFV wall thickness in differentiating BD patients from both CD and UC.

## MATERIALS AND METHODS

### Study population

In this cross-sectional study, patients with BD classified according to International Study Group (ISG) criteria (n=117), patients with CD (n=53), and UC (n=34) diagnosed according to the European Crohn’s and Colitis Organisation guidelines that are followed up at Rheumatology and Gastroenterology clinics in Marmara University Hospital were included.^3,15^ Healthy controls (HC) (n=85) whose age and gender were matched with the disease groups and with no history of chronic disease were selected among hospital staff and participated in the study.

The clinical characteristics of BD and IBD patients were recorded during routine follow-up visits. Montreal classification was used to define the characteristics of IBD. Crohn’s Disease Activity Index (CDAI), partial MAYO clinical score, and Behçet Syndrome Activity Score (BSAS) were used to assess the disease activity.

The Marmara University Ethical Committee approved the study protocol (No: 09.2020.373), and all participants provided written informed consent. The study performed following the latest amendments of the Declaration of Helsinki principles.

### Venous Doppler Ultrasonography

An experienced radiologist performed lower extremity venous Doppler US on the day of the clinical assessment during the routine follow-up visit of the patients. Bilateral common femoral veins were examined by a Doppler ultrasound (İu22 Philips Health Care, Bothell, WA, USA) with a high-resolution linear transducer (8–12 MHz). The veins were assessed from cranial to caudal direction. The wall thickness of CFV was measured using B-mode ultrasound while the participants were in the supine position. The wall thickness measurements were done at 2 cm distally from the saphenofemoral junction and from the posterior wall of CFV, to avoid reverberation artifacts that can be seen during the examination of the anterior wall. The mean of two consecutive measurements was recorded.

### Statistical analysis

SPSS version 22.0 (IBM Corp, Armonk, NY) was used for statistical analysis. The results were presented as frequency (%) for categorical data, mean and standard deviation for parametric data, and median and interquartile range (IQR) for skewed data. Comparison analysis of two continuous variables was made by using the Mann-Whitney U test or independent sample t-test according to data distribution. The Kruskal-Wallis test or one-way ANOVA was used to compare more than two continuous variables, and post-hoc analyses of more than two independent variables were made with the pairwise comparisons or Dunn–Bonferroni post-hoc test, respectively. The p-values for post-hoc analysis were given with Bonferroni correction where appropriate. The correlation between nonparametric continuous data was assessed by using Spearman’s rank-order correlation. The comparison of categorical variables was made using Fisher’s exact test or chi-square test. A *p-value* of less than 0.05 was considered statistically significant.

## RESULTS

### Patient and disease characteristics

Gender, body mass index (BMI), and smoking rates were comparable in all groups. The age of IBD patients was significantly higher than BD patients and HC (p<0.001). The clinical characteristics of the patients are given in **Table 1**.

**Table 1.** Demographic and clinical characteristics of the study groups.

	**Behçet's Disease^a^ (n=117)**	**Crohn's disease^b^ (n=53)**	**Ulcerative colitis^c^ (n=34)**	**Healthy controls^d^ (n=85)**	**p value**

**Age,** years, (mean±SD)	35.0 ± 8.2	42.2 ± 12.9	43.6 ± 14.5	32.3 ± 7.2	a*b<0.001
					a*c<0.001
					a*d = 0.37

**Gender,** male, n (%)	75 (64.1)	28 (52.8)	20 (58.8)	59 (69.4)	0.91

**BMI,** kg/m^2^, (mean±SD)	26.3 ± 7.3	25.3 ± 5.2	27.7 ± 5.1	24.0 ± 2.6	0.29

**Smoking rate,** n (%)	40 (34.2)	20 (38.1)	8 (23.5)	14 (36.8)	0.15

**Disease duration,** months, median (IQR)	97.0 (48.0–159.0)	90.0 (29.5.0–111.5)	106.7 (48.0–144.0)	-	0.06

**Immunosuppressive use,** n (%)	70 (59.8)	44 (83.0)	9 (26.4)	-	0.78
**Azathioprine**	66 (56.4)	26 (49.1)	8 (23.5)		
**Biological treatment**	16 (13.7)	33 (62.3)	3 (8.8)		
**Corticosteroids**	6 (5.1)	4 (7.5)	0 (0)		

BMI: body mass index; CFV: common femoral vein; IQR: interquartile range; SD: standard deviation.

a One-way ANOVA.

b Adjusted p values in pairwise comparisons with Bonferroni correction were 0.375 for BD vs. HC, 1.000 for CD vs.UC, and <0.001 for BD vs. CD, BD vs. UC, HC vs CD, and HC vs. UC.

Among BD patients, 70 (59.8%) had major organ (48[41.0%] vascular, 21[30.0%] ocular, 11[15.7%] gastrointestinal, and 8[11.4%] neurological) involvement. All BD patients with major organ involvement were using immunosuppressive (IS) treatment, and the remaining BD patients were using colchicine. The mean BSAS for BD patients was 26.9 ± 17.3.

Disease extension of UC was proctitis in 16 (47.1%), extensive colitis in 10 (29.4%), and left-sided colitis in 8 (23.5%) patients. Nine (26.5%) UC patients were using IS treatment, and all UC patients except one were using 5-ASA. The mean partial Mayo score for UC disease activity was 2.6 ± 1.5, and 16 (47.1%) patients had moderate or severe disease. Disease location of CD patients was ileocolonic in 30 (56.6%), ileal in 18 (34.0), colonic in 5 (9.4%) patients, and the most common behaviour type was non-stricturing non-penetrating (41.5%). Mean CDAI was 110.9 ± 76.3, and 14 (26.4%) of the CD patients had active disease (CDAI>150). Forty-four (83.0%) patients with CD were using an IS treatment. Extraintestinal involvements were oral aphthous ulcers in 19 (22.1%), erythema nodosum in 5 (5.8%), arthritis in 22 (25.6%), and uveitis in 2 (4.0%) IBD patients. Oral ulcers, erythema nodosum, and uveitis were present at significantly higher rates in BD patients (p<0.001, p<0.001, p=0.02, respectively), while the frequency of arthritis was similar to IBD. (p=0.49).

### Measurement of the wall thickness of common femoral vein

**Figure 1.**
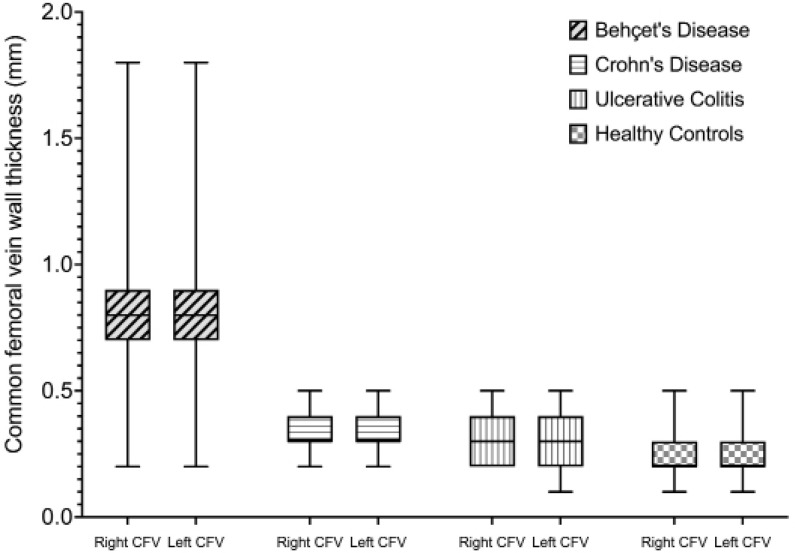
Distribution of the thicknesses of common femoral vein in study groups.

Mean right CFV thickness was 0.75 ± 0.21 mm, 0.32 ± 0.08 mm, and 0.28 ± 0.13 mm for BD, IBD, and HC, respectively. Mean left CFV thickness was 0.76 ± 0.21 mm, 0.32 ± 0.09 mm and 0.28 ± 0.13 mm for BD, IBD, and HC, respectively. Bilateral CFV thicknesses were significantly higher in BD compared to IBD patients and HC (adj. p <0.001 for both). CFV wall thicknesses in IBD patients were comparable to HC (adj. p >0.05 for both). Measurements of CFV wall thicknesses are shown in **Table 2** and **Figure 1**.

**Table 2.** The measurements of common femoral vein thickness.

	**Behçet's Disease (A) (n=117)**	**Inflammatory Bowel Disease (B) (n=87)**	**Healthy controls (C) (n=85)**	**p value^a^**	**Post-hoc test^b^**

**Right CFV thickness, mm** (mean±SD)	0.75 ± 0.21	0.32 ± 0.08	0.28 ± 0.13	<0.001	A vs. B, <0.001
					A vs. C, <0.001
					B vs. C, 0.060

**Left CFV thickness, mm,** (mean±SD)	0.76 ± 0.21	0.32 ± 0.09	0.28 ± 0.13	<0.001	A vs. B, <0.001
					A vs. C, <0.001
					B vs. C, 0.067

CFV: common femoral vein; SD: standard deviation.

a Kruskal Wallis test.

b Adjusted p values in pairwise comparisons with Bonferroni correction.

BD patients with and without major organ involvement had similar bilateral CFV wall thicknesses (p=0.53 for the right, p=0.21 for the left). CFV wall thicknesses were also comparable in BD patients with and without GI involvement (p=0.64 for right, p=0.27 for left).

Among IBD patients, bilateral CFV thicknesses were similar in patients with CD (0.33 ± 0.06 mm) and UC (0.30 ± 0.09 mm) (p=0.13 for right, p=0.14 for left).

There was no significant relationship between the wall thickness of CFV and age, gender, and BMI (p=0.90, p=0.89 and p=0.46, respectively).There was no significant relationship between IS use and CFV wall thicknesses in both BD and IBD groups (p=0.53 for right and p=0.21 for left in BD group, p=0.16 for right and p=0.41 for left in IBD group). Additionally, when BD and IBD patients using IS treatment were compared, CFV wall thicknesses were significantly higher in BD patients than in IBD patients (p<0.001 for right and left). Moreover, there was no difference in CFV thicknesses between the patients with active or inactive disease in both BD and IBD groups (p=0.21 and p=0.98). Disease duration also did not correlate with CFV thicknesses in both BD (p=0.62) and IBD patients (p=0.051).

## DISCUSSION

The BD diagnosis might be difficult in patients presenting with GI symptoms who do not meet the ISG criteria^15^, especially in regions with a low BD prevalence. We previously showed that the CFV wall thickness is increased in BD patients compared to other inflammatory diseases (ankylosing spondylitis, antiphospholipid syndrome) and CD.^9,14^ In this study, we found significantly higher CFV thickness in BD patients than in both CD and UC patients with similar results between IBD patients and HC.

GIBD diagnosis is mainly made on the presence of ulcerative intestinal lesions accompanied by the characteristic findings of BD and the exclusion of other reasons for intestinal inflammation including the use of certain drugs, infections, and IBD. However, typical BD manifestations are not always present in patients presenting with GI symptoms. In a study by Jung HC et al., nearly 30% of the patients with ileocolonic ulcers and no BD-related clinical findings at presentation were subsequently diagnosed as BD after 38 months of follow-up.^16^ Moreover, clinical findings like oral aphthous ulcers, erythema nodosum, and uveitis can be seen in both BD and IBD patients, and skin pathergy test might be positive in nearly 10% of CD patients.^17^

Intestinal lesions in BD have no pathognomonic gross features or specific pathological findings. It was reported that round oval ulcers were the most commonly seen intestinal lesions in GIBD patients.^6^ In another study, a classification including the clinical features, shape, and distribution of the ulcers was proposed, and it was reported that focal involvement and round ulcers were associated with BD, while diffuse or segmental involvement and longitudinal ulcers were associated with CD. However, these proposed criteria were based mainly on expert opinion and may not represent other regions with a low prevalence of GIBD.^18^

The characteristic vasculitis histology leads to a diagnosis of GIBD, but it is not frequently found in the biopsies.^19^ Histologic examinations mostly show a nonspecific inflammatory infiltrate around ulcers surrounded by normal intestinal mucosa, which can also be seen in IBD.^20^ Therefore, histologic examinations are mostly useful in excluding other intestinal diseases.

Moreover, there is no specific laboratory marker for the diagnosis of GIBD. Fecal calprotectin is commonly used in gastroenterology clinical practice to differentiate IBD from noninflammatory disorders, but it was also reported to be high in GIBD patients.^21–23^ Anti-*Saccharomyces cerevisiae* antibodies (ASCA) were found in high levels in both IBD and GIBD patients.^24,25^ In a meta-analysis an association between ASCA and GIBD is reported, but it is not useful to distinguish GIBD from intestinal tuberculosis.^26^ In a Chinese cohort, zymogen granule glycoprotein GP2 (aGP2) antibodies were found to be more efficient than ASCA in terms of distinguishing CD from GIBD and intestinal tuberculosis.^27^ However, these findings need to be validated in different populations.

In a study, it was shown that miR-195, miR-424, miR-10b, miR-103a-3p, and miR-542-3p markers are elevated in BD patients compared to HC, and miR-195 was found to be higher in vascular BD patients.^28^ Also, recent studies have shown that genetic factors, including miRNA dysregulation, play a major role in the pathophysiology of IBD, and multiple miRNAs participate in the complex regulatory system of intestinal inflammation.^29^ However, since no studies have compared BD and IBD patients in this regard, there is no clear data on the use of these markers in distinguishing between BD and IBD.

In BD, vascular involvement is seen in both veins and arteries, most commonly as deep venous thrombosis. Although IBD patients also have a high risk for venous thrombosis, we did not find any tendency for increased venous wall thickness in our study. It is suggested that increased wall thickness of CFV in BD patients might be a sign of venous inflammation. In our study, we found that BD patients had higher CFV wall thickness compared to IBD patients, independent of IS use. Although the rate of biological treatment use was higher in CD patients, IS use within the IBD group did not affect CFV wall thickness. Moreover, our previous studies have shown that CFV wall thickness in BD patients is higher than in healthy controls, regardless of treatment. While these results suggest that IS treatments may not have a significant impact on CFV wall thickness, studies with treatment-matched cohorts may be needed to obtain more conclusive findings.

The main limitations of this study are the small sample size of UC patients compared to other groups and the absence of inter-observer reliability analysis. However, we have previously reported good inter-observer reliability of CFV thickness measurement in our Unit.^9,30^

In conclusion, we found higher CFV wall thickness in BD patients compared to IBD patients and HC. These results may suggest that the measurement of CFV wall thickness, which is a non-invasive and accessible method, may be used to differentiate BD from IBD in patients with obscure clinical features in clinical practice.

## FUNDING

None.

## CONFLICT OF INTEREST

The authors have no conflict of interest.

## AUTHOR CONTRIBUTIONS

**GS:** Design, supervision, data collection, analysis and interpretation, literature review, writing, critical review; **RE:** Design, data collection and interpretation, writing, critical review; **IE:** Design, data collection and interpretation, literature review, writing, critical review; **ES:** Design, data collection and interpretation, critical review; **FT:** Data collection, critical review; **OA:** Design, supervision, data collection, analysis and interpretation, literature review, writing, critical review; **HD:** Design, supervision, data collection, analysis and interpretation, literature review, writing, critical review; **FAO:** Design, supervision, data collection, analysis and interpretation, literature review, writing, critical review.
